# Synaptic, cellular, and circuit mechanisms underlying sex differences in behaviors

**DOI:** 10.1186/s13293-026-00931-8

**Published:** 2026-05-29

**Authors:** Ipe Ninan

**Affiliations:** Department of Neurosciences and Psychiatry, University of Toledo, Toledo, 43614 OH USA

**Keywords:** Sex differences, Synapses, Neuronal subtypes, Organizational effects, Neural circuitry, Activational effects, Sex chromosomes, Sex hormones

## Abstract

Consistent with differences in behaviors between sexes, studies reveal sex differences in the organization of neural circuits, synaptic function, and neuronal excitability, as well as sex-dependent recruitment of specific neuronal subtypes during behavior. These studies demonstrate sex differences in cell numbers, brain region volumes, cellular composition of brain areas, and density and strength of synapses in many brain regions, innervation of neuronal subtypes, receptor-mediated transduction mechanisms, neurotransmitter and neuropeptide release, and the influence of neuronal growth factors. Beyond insights into the mechanisms of sex differences in behaviors, understanding sex-typical circuit, cellular, and synaptic processes is crucial for identifying the causes of sex-typical vulnerabilities to nervous system disorders, such as the high prevalence of autism spectrum disorders and attention deficit hyperactivity disorder in males, and the higher incidence of affective, anxiety, and trauma-related disorders in females. Because these disorders often emerge during various developmental stages, it is essential to understand how development interacts with genetic, epigenetic, metabolic, hormonal, and environmental factors to affect the nervous system across sexes. Consequently, future research that examines the interaction between the nervous system and these critical factors is expected to elucidate the mechanisms underlying nervous system disorders. Additionally, targeting sex-specific mechanisms involved in these disorders could open new opportunities for more effective treatments in both sexes.

## Highlights

Sex-dependent recruitment of specific neurons in behavior.

Sex differences in synaptic function and neuronal excitability.

Sex-specific organization of neural circuits.

Sex differences in synaptic, cellular, and circuit effects are mediated by the organizational effects of sex chromosomes and sex hormones, as well as the activational effects of sex hormones.

Recent advances in sex differences in the nervous system and behavior lay a foundation for future studies to reveal sex-specific mechanisms in behavior and nervous system disorders.

## Background

For a long time, studies on sex differences in nervous system organization, sex-typical engagement of brain regions and neuronal subtypes, and their effects on behavior have been very limited. Recent interest in sex as a biological variable, along with new data, has drawn significant attention to sex differences in neural circuitry, cellular mechanisms, synapses, and their involvement in behavior. Consistent with sex-exclusive behaviors and differences in behavioral strategies and in the magnitude of various behaviors between females and males, sex differences have been observed in the organization of brain structures, synaptic function, and the involvement of specific neuronal subtypes in behavior. The sex difference in brain organization could be attributed to sex hormones and sex chromosomes, including the presence of Y chromosomal genes in males and dosage differences in some X chromosomal genes in females and males due to escape from X chromosome inactivation [1–6]. In addition to the effects of chromosomal, hormonal, and environmental factors during development on the sex-typical organization of the nervous system, fluctuating hormone levels are expected to dynamically modulate neuronal function, thereby influencing behavior. Therefore, sex differences in behaviors are mediated through sex-typical establishment of synapses and neuronal circuits, the dynamic effects of molecular signaling involving hormones, neurotransmitters, and neuropeptides, or differences in gene expression [7]. Besides driving sexually dimorphic or exclusive behaviors, these mechanisms might also create quantitative differences in specific behaviors between females and males. The terms “sex-specific”, “sex-typical”, and “sex differences” are used throughout this review to denote a characteristic occurring in only one sex, more common in one sex, and group-level differences, respectively.

Sex differences in the organization of the nervous system are evident in human studies. Apart from the higher brain mass in males than in females, studies show sex differences in various brain structures [8]. For example, the number of neurons in the occipital cortex is higher in men than in women, whereas women show higher neuronal density in the frontal cortex (8). Males show a larger amygdala, but studies on the hippocampus show no conclusive size difference between women and men [9, 10]. Males exhibit greater within-hemispheric connectivity, while females show predominant inter-hemispheric connectivity, suggesting possible mechanisms behind sex differences in motor and spatial abilities, memory, and social cognition [11]. During development, males exhibit greater variability in brain volumes compared to females [12]. However, males tend to show greater homogeneity across regional brain volumes than females [12]. With advances in experimental tools, future research is expected to uncover additional significant differences, including differences at the cellular and synaptic levels, in male and female brains. While identifying sex differences in the human nervous system is important for understanding behavioral differences between females and males and sex-dependent vulnerabilities to nervous system disorders, advanced animal studies are necessary to clarify the mechanisms underlying sex differences in behavior and associated disorders.

**Fig. 1:**
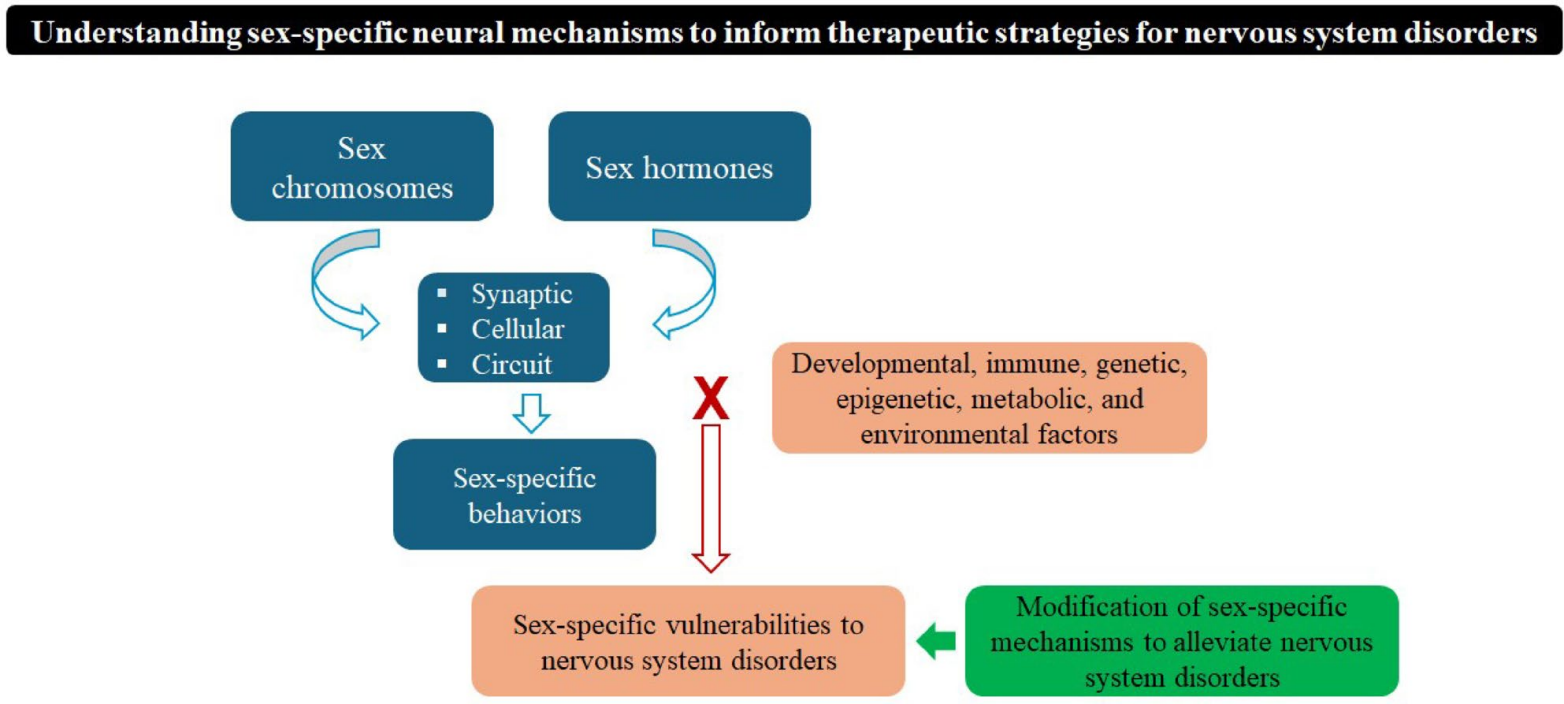
The effect of sex chromosomes and sex hormones on synaptic, cellular, and circuit mechanisms guides sex-specific behaviors. The interaction of these mechanisms with developmental,immune, genetic, epigenetic, and environmental factors might lead to sex-specific vulnerabilities to nervous system disorders. Therefore, understanding the mechanisms involved in sex-specific behaviors might provide opportunities for developing therapeutic strategies to alleviate nervous system disorders

To advance the field of mechanisms underlying sex differences in behaviors and to inform the development of future therapeutic strategies for nervous system disorders, it is crucial to appraise the current understanding of the roles of specific neuronal subtypes, synapses, and brain circuits in behavior in both sexes (Fig. 1). Consistent with many sex-typical behaviors that facilitate reproduction, parental care, resource acquisition, social network establishment, and protection against threats, which are essential for the survival of the species, recent studies have begun to elucidate the cellular, synaptic, and circuit mechanisms underlying these sex-typical behaviors. Unlike human studies, which are limited by technical constraints and preclude in-depth investigation of the mechanisms underlying sex differences in behaviors, animal studies have provided significant insights into the specific roles of cell types, synapses, and molecular mechanisms in behavior in females and males. Studies show that specific cell types mediate sexually dimorphic behaviors, suggesting sex-typical recruitment of particular neuronal cell types in the regulation of behavior [13, 14]. In addition, synapses, membrane receptors, and ion channels, along with their downstream signaling pathways, play a critical role in sex differences in behaviors [15, 16]. Effects of sex hormones on synapses and neuronal excitability suggest their dynamic role in shaping behavioral sex differences [16, 17]. Consistently, neurons in many brain regions exhibit sex differences in synaptic responses that depend on sex hormone levels [16, 18]. Another key neural mechanism underlying behavioral differences between females and males is sex differences in the organization of brain structures, with studies demonstrating sex differences in volume, cellular distribution, and cellular density, as well as in the roles these mechanisms play in sex-typical behaviors.

Therefore, this review summarizes the sex-dependent roles of specific neurons, sex differences in synaptic function, and sex-typical organization of brain circuits, and their relevance to behavior.

### Sex differences in the recruitment of neuronal subtypes in behavior

Studies show that engagement of specific neuronal subtypes in behavior differs between sexes. For example, there are sex differences in the behavioral effects of activating oxytocin receptor-expressing gamma-aminobutyric acid (GABA)ergic neurons in the medial prefrontal cortex [19]. This effect is evident at the single-cell level, as oxytocin preferentially enhances action potentials in oxytocin receptor-expressing GABAergic neurons in the medial prefrontal cortex of female mice, compared with male mice [19]. Consistently, oxytocin receptor-expressing interneurons in the medial prefrontal cortex play a crucial role in male-directed social behavior in females during the sexually responsive phase of the estrous cycle [19]. Oxytocin receptor-expressing interneurons in the medial prefrontal cortex mediate anxiolytic effects in male but not female mice by releasing corticotropin-releasing hormone binding protein, which blocks the effect of corticotropin-releasing hormone (CRH) [20]. The male-specific effect of oxytocin receptor neurons on anxiety also involved heightened sensitivity of layer 2/3 pyramidal neurons to CRH, which was blocked by corticotropin-releasing hormone-binding protein. This sex-specific effect mediated by oxytocin receptor-expressing neurons in the cortex might enable sex-typical regulation of downstream brain regions. Unlike oxytocin, which is released from hypothalamic neurons, medial prefrontal cortical 5-HT3aR-expressing neurons release CRH [21]. These CRH-expressing neurons are connected to oxytocin receptor-expressing neurons and layer 2/3 pyramidal neurons through inhibitory synapses [21]. Interestingly, this synaptic connectivity is significantly higher in males compared to females [21]. Similarly, optogenetically induced release of CRH elicits sex differences in responses in layer 2/3 pyramidal cells [21]. Furthermore, activation of local CRH release in the medial prefrontal cortex modulates novelty exploration in males without affecting anxiety behaviors in males or females [21]. Thus, oxytocin receptor neurons and CRH-expressing neurons mediate sex differences in social, novelty, and emotional behaviors through distinct mechanisms in the medial prefrontal cortex.

The sex-dependent role of dorsal medial prefrontal cortical parvalbumin GABAergic neurons has been demonstrated in social hierarchy and winner effect [22, 23]. Female mice show higher excitability in dorsal medial prefrontal cortical parvalbumin-expressing interneurons, which negatively regulate thalamo-cortical plasticity involved in the ability to form social hierarchies and winner effect [24]. Neurons in the bed nucleus of the stria terminalis (BNST), referred to as the extended amygdala, and the lateral habenula that express 5-HT2C receptors show sex differences in their role in rewarding and aversive behaviors [25]. BNST 5-HT2C neurons in males show higher activation during social interaction and in response to acoustic stimuli than in females [25]. In contrast, these neurons in males show higher inhibition in response to alcohol and water consumption [25]. Unlike males, 5-HT2C neurons in females exhibit a differential response to water and alcohol consumption [25]. The BNST 5-HT2C neurons in females play a predominant role in reward consumption compared to males [25]. Similarly, studies show that specific neuronal subtypes can play a role in sex-specific impairment in cognitive performance [26].

Recent studies show sex differences in the neuromodulation of the forebrain. For example, the median raphe nucleus modulates the medial prefrontal cortex and the hippocampus by activating local GABAergic neurons in a sex-dependent manner [27, 28]. The vesicular glutamate transporter 3 (VGLUT3)-expressing median raphe neurons play an important role in forebrain functions [27–29]. Activation of median raphe VGLUT3 neurons suppresses fear memory only in females, and this behavioral effect involves a selective enhancement of GABAergic transmission and a suppression of glutamatergic transmission in the prelimbic medial prefrontal cortical pyramidal neurons of female mice [27]. On the other hand, activation of median raphe VGLUT3 neurons selectively enhances GABAergic transmission onto dorsal hippocampal CA1 pyramidal neurons in male but not female mice [28]. This sex-specific effect on GABAergic transmission also leads to selective impairment of hippocampal plasticity in males [28]. Similarly, dopamine neurons in the ventrolateral periaqueductal gray and dorsal raphe selectively mediate pain suppression in males while selectively promoting locomotion in females through dopaminergic transmission to the BNST [30]. These findings are significant given the disproportionate experience of pain in females and our lack of insights into the underlying mechanisms [31]. The ventral tegmental area (VTA) dopaminergic neurons are sex-specifically modulated by stress. Subchronic stress suppresses tonic firing of VTA dopaminergic neurons in females but not males, as males show higher glutamatergic input to these neurons, a likely mechanism that offsets the suppression of tonic firing [32]. These sex-specific effects in dopaminergic neurons might contribute to sex differences in stress responses. Similar to dopamine neurons, sex differences are observed in serotonin modulation of neurons. Serotonin release on 5-HT2C-expressing BNST neurons is greatly reduced in females compared to males during water and alcohol consumption [25]. It was also shown that the experience of social isolation affects the excitability of serotonin neurons in a sex-specific manner, suggesting that targeting serotonin signaling to reverse stress-induced effects requires a sex-based approach [33]. The serotonergic neurons in females are less excitable than in males, suggesting a potential role of neuronal excitability in permitting sex-specific recruitment of neurons in behavior [34]. In addition, sex differences are observed in serotonin neuron responses to CRH, further supporting the sex-typical role of serotonin neurons in stress responsivity and their potential role in the high incidence of affective disorders in females [34]. Sex-typical expression of serotonin receptors also might contribute to sex differences in serotonin neuromodulation [35]. Serotonin regulates dopamine-dependent cortico-striatal plasticity in a sex-specific manner [36]. As with dopamine and serotonin, the release of norepinephrine from the brainstem nucleus locus coeruleus plays a critical role in stress-related behaviors, with sex-typical effects. Females show increased dendritic density in locus coeruleus neurons [37], a potential mechanism for enhanced response to synaptic inputs associated with stress-related behaviors. Females also show higher stimulation of locus coeruleus neurons following hypotensive stress [38]. In addition, locus coeruleus neurons in females exhibit higher sensitivity to CRH compared to males [38]. It has been shown that females, not males, recruit the noradrenergic system during active defense coping to interoceptive and exteroceptive threats [39]. Thus, sex differences in neuromodulation mediated by serotonin, dopamine, and norepinephrine, along with their interaction with signaling mechanisms associated with stress, might be a key factor in sex differences in stress-related disorders, anxiety, and affective disorders.

Given the high incidence of anxiety- and fear-related disorders in females, understanding sex differences in the amygdala, a brain region involved in mediating fear, is of great significance [40, 41]. Neurons in the lateral amygdala show higher activity in female rats compared to males [18]. This increased activity is influenced by endogenous estradiol levels, with higher activity during low-estradiol stages than during high-estradiol stages [18]. Although the basal nucleus of the amygdala shows a similar sex difference in neuronal activity, the effect of endogenous estradiol was the opposite, as low estradiol stages showed lower neuronal activity compared to high estradiol stages [18]. However, the mechanism by which endogenous estradiol exerts this region-specific differential modulation of neuronal activity is unclear. The sex difference in neuronal activity in both the lateral and basal nuclei coincided with the number of dendritic spines in females [18]. Therefore, estradiol might play a role in regulating sex-typical neuronal engagement across brain regions, leading to sex differences in behaviors. The BNST is a sexually dimorphic structure located in the basal forebrain. Interestingly, the BNST is involved in the expression of contextual fear memory in males but not in females [42]. The BNST shows sex differences in its neurochemical profile and volume [43–45]. The female anterolateral BNST contains more neurons, including CRH-expressing neurons [46, 47]. Also, knockdown of the serotonin 5-HT1A receptor in the BNST selectively enhances contextual fear memory expression in males, and this effect coincides with increased excitability in BNST neurons [48]. Expression of contextual fear memory involves upregulation of the immediate early genes ARC and c-Fos in male BNST neurons projecting to the central amygdala, but not in females, suggesting a sex-specific engagement of the BNST-central amygdala pathway in contextual fear memory [49]. This study also suggests that a different subtype of BNST neurons is responsible for contextual fear memory in females [49]. Given the role of the amygdala and BNST in anxiety and fear-related behaviors, future studies are needed to understand how these sex differences contribute to the high incidence of anxiety and fear-related disorders in females.

**Fig. 2:**
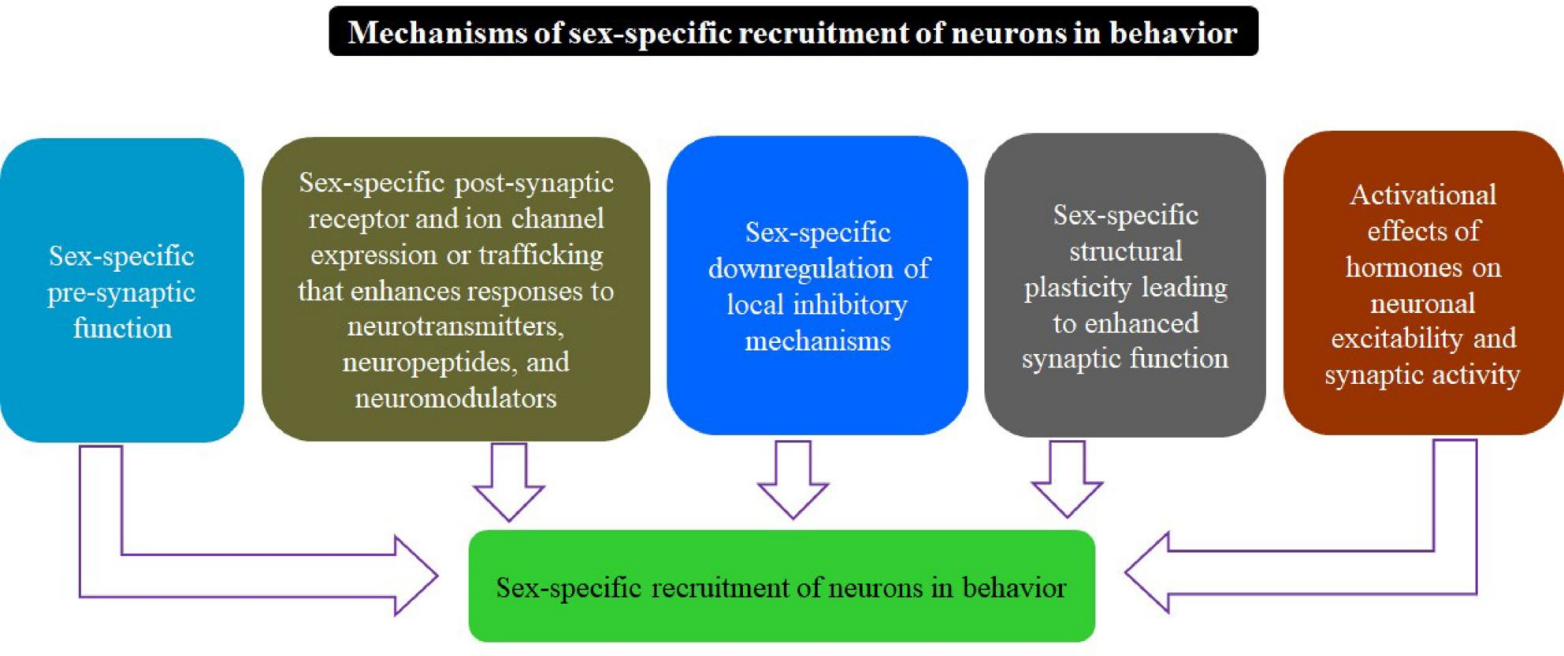
The sex-specific recruitment of neurons in behavior might involve sex-dependent pre-synaptic and post-synaptic function, local inhibitory mechanisms, structural plasticity, and the activational effects of hormones on neuronal excitability and synaptic function

While these studies show remarkable differences in the roles of neurons in behaviors between the sexes, further research is needed to understand how the sex-dependent engagement of specific neurons translates into sex-typical behaviors. In addition, it is crucial to understand the mechanisms that enable sex-typical engagement of neurons in behavior. Mechanisms such as sex-typical expression of receptors and ion channels that are activated by neurotransmitters, neuropeptides, and neuromodulators released by upstream brain regions, sex-typical organization of upstream synaptic inputs, leading to sex differences in pre-synaptic function, sex-specific downregulation of local inhibitory mechanisms, sex-specific structural plasticity that permits modification of dendritic spine density, dendritic branching and length, axonal growth, and number of synapses, and the dynamic effects of sex hormones on neuronal excitability and synaptic function might play a role in sex differences in the recruitment of neurons in behavior (Fig. 2). Thus, sex differences at both sides of the synapse are a major mechanism that permits differences in the engagement of specific neuronal subtypes in behavior in females and males. Consistent with the potential role of synapses in sex-dependent recruitment of neurons in behavior, studies have demonstrated notable differences in synaptic function between females and males.

### Sex differences in synaptic function and behavior

Sex differences in synaptic function are mainly reflected in synaptic density and molecular organization [50]. Other factors, such as the size and location of synapses, may also influence differences in synaptic responses between females and males. The sex-typical organization of synapses and its relevance to behavior are evident in vertebrates and invertebrates [16, 50, 51]. In addition to sex differences in the organization of synapses due to sex-chromosomal and sex-hormonal effects, synapses are dynamically influenced by fluctuations in sex hormone levels.

Given the sex difference in threat-related behaviors and their relevance to the high incidence of anxiety and affective disorders in females, many studies have addressed sex differences in synapses in brain areas relevant to the regulation of fear and threat behaviors [40, 41]. Given the role of ventromedial prefrontal cortex in fear extinction, an inhibitory learning that attenuates conditioned fear and is implicated in anxiety behaviors, an extensive analysis of synaptic transmission in the ventromedial prefrontal cortex pyramidal neurons during high- and low-estradiol stages in mice showed that low estradiol stages show higher alpha-amino-3-hydroxy-5-methyl-4-isoxazolepropionic acid (AMPA) receptor transmission [16, 52]. An analysis of N-methyl-D-aspartate (NMDA) receptor transmission showed that during low-estradiol stages, NMDA receptor activity is altered, with GluN2B subunit-mediated transmission reduced in ventral medial prefrontal cortical pyramidal neurons [16]. Consistent with these results, low-estradiol mice show diminished GluN2B-dependent plasticity in the ventral medial prefrontal cortex, suggesting specific synaptic mechanisms that might underlie reduced fear extinction in females during low-estradiol stages [16, 53]. Given the role of neurotrophins in the effect of estradiol, an altered neurotrophin activation downstream of estradiol signaling might be responsible for the diminished GluN2B-dependent plasticity [54, 55]. Estrogen receptor beta activation modulates AMPA receptor transmission in the ventral medial prefrontal cortex in an endogenous estradiol-dependent manner, i.e., suppressing and enhancing glutamatergic transmission, respectively, during low- and high-estradiol stages [16]. Importantly, comparable ventromedial prefrontal cortex plasticity in high estradiol females and males supports the suggestion that endogenous estradiol might play a critical role in sex differences in fear extinction [16, 53]. Thus, endogenous estradiol-dependent plasticity in the ventromedial prefrontal cortex and, hence, robust top-down regulation of the amygdala might be crucial for enhanced fear extinction during high-estradiol stages [56, 57]. It is also possible that fear extinction in females and males involves a differential modulation of downstream amygdala by the ventromedial prefrontal cortex [58]. A sex difference is also observed in glutamatergic synaptic transmission in the medial prefrontal cortex, with higher AMPA receptor expression in females [59]. The median raphe nucleus, a brain region that suppresses fear memory in females but not males, selectively enhances GABAergic transmission in prelimbic medial prefrontal cortical pyramidal neurons of females [27]. In addition, VGLUT3-expressing median raphe nucleus neurons respond to fear learning in a sex-specific manner, as only female median raphe neurons show enhancement of excitatory transmission after fear conditioning [27]. Also, the excitatory and inhibitory neurons in the prelimbic medial prefrontal cortex, a major target of VGLUT3-expressing median raphe neurons, exhibit sex-typical responses to activation of local VGLUT3-expressing neurons that release both glutamate and GABA [27]. These studies demonstrate robust sex differences in synaptic function within the medial prefrontal cortex, which plays a critical role in regulating anxiety and emotional behaviors that disproportionately affect females compared with males.

In addition to the medial prefrontal cortex, the amygdala, a brain area necessary for mediating fear behavior, exhibits sex differences in synaptic transmission. Female rats show higher glutamatergic transmission in the principal neurons of the lateral and basal amygdala compared to male rats, suggesting a potential role for glutamatergic synaptic inputs in sex differences in neuronal activity [18]. These studies also showed increased GABAergic synaptic input to lateral and basal amygdala principal neurons in females compared with males, suggesting that the excitatory-to-inhibitory ratio of synaptic inputs to these neurons might be critical for their sex-specific role in fear behaviors [18]. Basolateral amygdala neurons that project to the BNST mediate a male-specific anticipatory anxiety-like behavior, as these neurons exhibit higher excitability in males than in females [60]. Given the robust effect of estradiol signaling on sex-specific synaptic plasticity in the basolateral amygdala, future studies are needed to elucidate the mechanisms underlying the amygdala’s sex-specific role in behaviors, particularly fear- and anxiety-related behaviors [61].

Similarly, in the hippocampus, sex differences in synaptic transmission have long been recognized. Despite having fewer dentate gyrus granule cells, females show a higher density of synapses on CA3 pyramidal cells than males [62]. The hippocampus plays a critical role in contextual fear memory [63]. Consistent with higher contextual fear learning in males compared to females, both the long-term potentiation (LTP) and NMDA receptor activity at the perforant path-dentate gyrus synapses of the hippocampus are diminished in females compared to males [64]. Studies also show consistently higher spatial learning and memory performance and robust perforant pathway-dentate gyrus LTP in males compared to females [65]. Chronic intermittent theta-burst stimulation increases the size of mossy fiber terminals that synapse onto CA3 pyramidal neurons in male but not female mice, suggesting sex differences in hippocampal structural plasticity in response to activity [66]. Sex difference in Schaffer collateral-CA1 LTP in the hippocampus was also reported [28, 67]. These findings are supported by other studies showing diminished contextual fear learning in females [68, 69]. Studies also show that synaptic inputs from other brain regions modulate hippocampal synaptic transmission and plasticity in a sex-specific manner. Activation of median raphe VGLUT3 neurons enhances GABAergic transmission onto CA1 pyramidal neurons and selectively suppresses LTP in males, suggesting that the median raphe glutamatergic input might selectively modulate hippocampus-mediated behaviors in males [28]. Studies suggest that estradiol plays a key role in sex-specific effects on synapses in the hippocampus. Estradiol selectively suppresses GABAergic transmission in the hippocampus of female mice [70, 71]. Although estradiol enhances glutamatergic transmission in the hippocampus of both females and males, this effect is mediated by distinct mechanisms involving different estrogen receptors in females and males [72]. Sex differences in mechanisms other than hormones could also affect hippocampal synapses. Endocannabinoids contribute to sex differences in hippocampal synaptic function [73]. Despite the correlation between behaviors and sex-specific synaptic activity in the hippocampus, future studies are needed to understand how these synaptic changes shape sex differences in hippocampus-dependent behaviors.

The aforementioned sex differences in synaptic transmission and plasticity in fear circuitry, comprised of the medial prefrontal cortex, the amygdala, and the hippocampus, are particularly interesting given the high incidence of anxiety and fear-related disorders in females compared to males. In healthy individuals, awareness of potential threats, often acquired through past experiences, plays a critical role in detecting future threats and taking appropriate safety measures. Suppressing the memory of these threats in safe environments is equally essential for enabling other survival functions. However, anxiety and psychological trauma-related disorders show a maladaptive recall of fear memory and fear responses when there are no apparent threats. Studies based on the Pavlovian fear conditioning paradigm and associated changes in the medial prefrontal cortex, the amygdala, and the hippocampus have provided significant insights into mechanisms, specifically, circuit, cellular, and synaptic processes underlying the regulation of fear behavior. Consistent with sex differences in synaptic function in these brain areas, many studies have shown remarkable sex differences in behaviors relevant to fear and the underlying mechanisms. Furthermore, as with the role of sex hormones, particularly estradiol, in synaptic function, estradiol signaling also plays a critical role in augmenting fear extinction, a key mechanism in relieving anxiety behavior [53]. These findings lay the foundation for future studies to inform evidence-based approaches for managing anxiety and fear-related disorders in both sexes. Similarly, despite the strong evidence for sex differences in hippocampal synaptic transmission, synaptic plasticity, presynaptic machinery, neurogenesis, and dendritic complexity, which are highly relevant to cognitive function, a direct role for these mechanisms in sex differences in cognitive behaviors and disorders associated with cognitive impairment has yet to be established [74–77]. Given the complex mechanisms underlying cognitive behaviors, future studies are needed to demonstrate how sex-specific mechanisms at the synaptic and cellular levels regulate sex-typical cognitive behaviors and, hence, their role in disorders involving cognitive dysfunction.

**Fig. 3:**
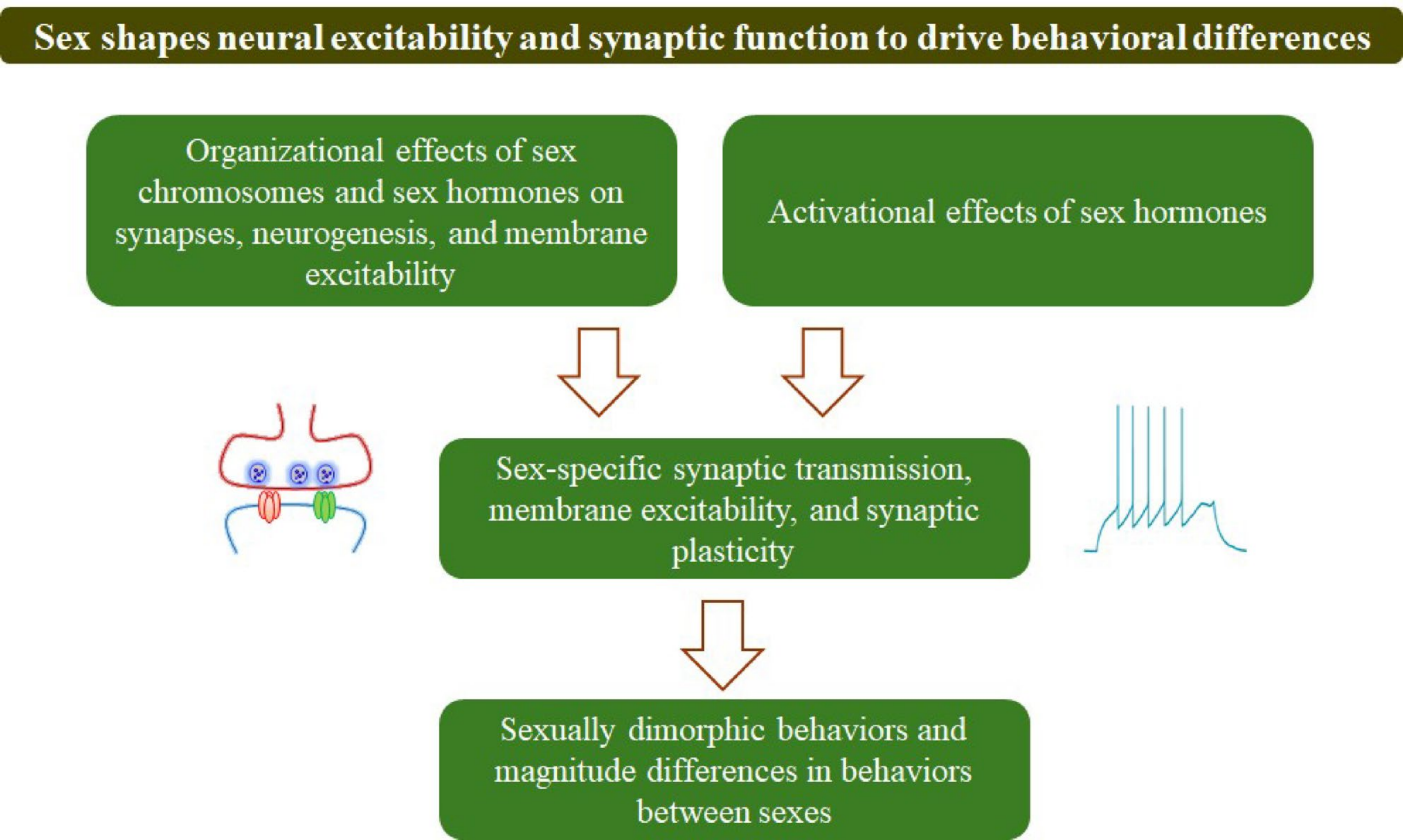
Sex differences in membrane excitability, synaptic transmission, and synaptic plasticity, driven by sex chromosomal and hormonal effects, play a critical role in sex differences in behavior

Similar to notable sex differences in synaptic properties, studies have shown sex differences in membrane excitability, a possible mechanism underlying sex-specific recruitment of neurons that mediates behavior [78, 79]. Thus, differences in synaptic properties and membrane excitability arising from the activational effects of sex hormones and the organizational effects of both sex chromosomes and sex hormones could play a critical role in sex-specific behaviors (Fig. 3). Beyond these specific sex differences in synaptic and cellular functions that affect behavior, sex chromosomes and sex hormones might exert a profound effect on the development of brain regions, leading to remarkable sex differences in the organization of brain circuitry, including neuronal density, regional size, and neural circuit architecture. These developmental effects play a critical role in sex-specific behaviors, including reproduction, parenting, and the regulation of emotional behavior.

### Sex-specific organization of brain circuitry and behavior

Sex-specific organization of brain circuitry plays a critical role in sex-typical behaviors. A robust example of sex differences in the organization of a brain area is the medial amygdala (MeA), as it has been well described in rodent studies. The MeA is a crucial player in social behavior, affecting aggression, parental care, and reproduction. The MeA, which is anatomically distinct from the amygdala regions involved in fear regulation, is located two synapses away from the vomeronasal organ (VNO), a sensory epithelium involved in pheromonal signaling [80, 81]. The VNO axons project to the accessory olfactory bulb (AOB), upstream of the MeA, and play a critical role in pheromone-mediated signaling and, hence, social behaviors [81]. Consistent with the sex difference in social behaviors, males show significantly higher volume of MeA compared to females [82]. The higher MeA volume in males is consistent with the increased dendritic branching in males compared to females [83]. In addition, neuron number and dendritic branching in the MeA are lateralized in males but not in females, suggesting potentially enhanced processing of MeA-mediated social behaviors that are critical in males [83]. In prepubertal male mice, the left hemisphere MeA exhibits more excitatory synapses than in age-matched female mice [84]. However, the number of GABAergic synapses is similar between females and males [84]. This higher MeA volume depends on androgen levels [82]. The MeA contains a significant number of aromatase-expressing neurons, suggesting aromatase-dependent conversion of testosterone to estrogen in the MeA [85]. Interestingly, males have more aromatase neurons than females [85]. The aromatase-induced conversion of testosterone to estradiol in males is critical for sex-specific development of the MeA and male-specific territorial behaviors [85–87]. The connectivity between AOB and the aromatase-expressing MeA neurons plays a critical role in social behaviors, including parenting, aggression, and mating [14, 88, 89]. Consistent with MeA-dependent sex-specific behaviors, the sex-typical organization of inputs to the MeA aromatase neurons has been demonstrated. For example, the synaptic convergence of AOB onto individual aromatase-expressing MeA neurons is significantly higher in males than in females [90]. While the MeA neurons respond strongly to the opposite sex stimuli in both sexes [91], the MeA response to predator stimuli is dominant in females [91]. This sexual dimorphism of sensory representation is mediated by the MeA [91]. The MeA-mediated sexually dimorphic responses depend on estrogen signaling, resulting from aromatase-induced conversion of testosterone to estrogen [91]. Apart from the early developmental steroid-dependent organization of the MeA circuit, further sex hormone-dependent circuit organization during puberty is necessary for the sexually dimorphic role of the MeA [91]. Although the adult MeA is significantly larger in males, the MeA cell proliferation is significantly higher in neonatal females [45, 92, 93]. In addition to the sex hormone-mediated organization of the MeA, suppression of endocannabinoid levels due to high expression of endocannabinoid-degrading enzymes maintains elevated cell proliferation in the female MeA, as endocannabinoids suppress cell proliferation via CB2 receptors, selectively in females [92]. Consistent with the notion that endocannabinoids mediate sex differences in MeA cell proliferation, cannabinoids masculinized juvenile play behavior in females without affecting males [92]. These effects are similar to those induced by neonatal estradiol or testosterone in females [94, 95].

Recent studies shed more light on the sex- and cell-type-specific functions of MeA in different social behaviors. MeA GABAergic neurons promote parental behavior in females, whereas in males, they influence both parental and infanticidal behavior, depending on their activity levels: high activity in MeA GABAergic neurons resulted in infanticidal behavior, whereas lower activity led to parental behavior [96]. This sexually dimorphic effect is not attributed to sex differences in cell types, neuronal subtype identity, or their relative abundance [96]. However, glutamatergic neurons in both females and males promote non-social self-grooming behavior [96]. Consistently, GABAergic neurons but not glutamatergic neurons in MeA show sex differences in gene expression [96]. These findings are in agreement with the role of MeA GABAergic projections in lordosis, a female-specific behavior, while activation of glutamatergic MeA neurons enhances non-social self-grooming behavior [97]. Aromatase-expressing MeA neurons, which are primarily GABAergic, play a key role in aggressive behaviors in males [14]. Similarly, maternal aggression in females depends on the activation of aromatase-expressing MeA neurons [14]. Among the different subtypes of MeA neurons, two different subtypes deriving from progenitor populations expressing Dbx1 and Foxp2 show sex differences in their activation during mating [98]. Furthermore, membrane properties and gene expression, particularly expression of ion channels involved in membrane excitability, show sex differences, suggesting that both cell-type-specific activation and intrinsic properties of MeA neurons play a critical role in MeA-mediated sex-specific behaviors [99].

Another brain area that shows remarkable sex-specific organization is the BNST, which is critical for stress-related responses, anxiety, and social behavior [100]. The BNST plays a key role in contextual fear memory [101]. Unlike the amygdala, which is critical for the expression of phasic fear, the BNST is believed to play a critical role in responses to unpredictable threats and in mediating sustained fear [100]. Similar to the MeA, males show a higher number of aromatase neurons in the BNST than females [85]. In addition, males show higher levels of axonal output from the aromatase-expressing neurons [85]. These studies suggest that the target cells are sex-specifically innervated by the aromatase-expressing neurons. As in the case of the MeA, the aromatase-mediated conversion of testosterone to estrogen is critical for BNST neuronal survival in males [85]. Consistent with the sex-specific organization of the BNST, the BNST is involved in contextual fear memory expression in males but not females [42]. The BNST shows sex differences in its neurochemical profile and volume [43–45]. The female anterolateral BNST shows a higher number of neurons, including CRH-expressing neurons [46, 47]. Also, the BNST shows sex-specific serotonin-dependent behaviors as knockdown of the serotonin 5-HT1A receptor in the BNST enhances contextual fear memory expression selectively in males, and this effect coincides with enhanced excitability in BNST neurons [48]. Expression of contextual fear memory involves upregulation of the immediate-early genes ARC and c-Fos in male BNST neurons that project to the central amygdala, but not in females, suggesting a sex-specific engagement of the BNST-central amygdala pathway in contextual fear memory [49]. This study also suggests that a different subtype of BNST neurons is responsible for contextual fear memory in females [49]. Thus, sex-specific circuit, synaptic, and intrinsic neuronal mechanisms contribute to the sex-dependent role of BNST in fear behavior.

Similar to the MeA and the BNST, the sexually dimorphic nucleus of the preoptic area (SDN-POA) exhibits robust sex differences in its organization, a feature consistent across multiple species [102]. The volume of SDN-POA is remarkably higher in males compared to females [102]. Studies show the role of higher apoptosis in females in the sex-specific development of SDN-POA [103]. The phagocytic microglia plays a critical role in establishing sex-specific organization of SDN-POA, and this developmental process depends upon the aromatase-mediated conversion of androgens to estrogens [104, 105]. Sexual dimorphism in the SDN-POA is thought to contribute to male-typical mating preferences [106].

**Fig. 4:**
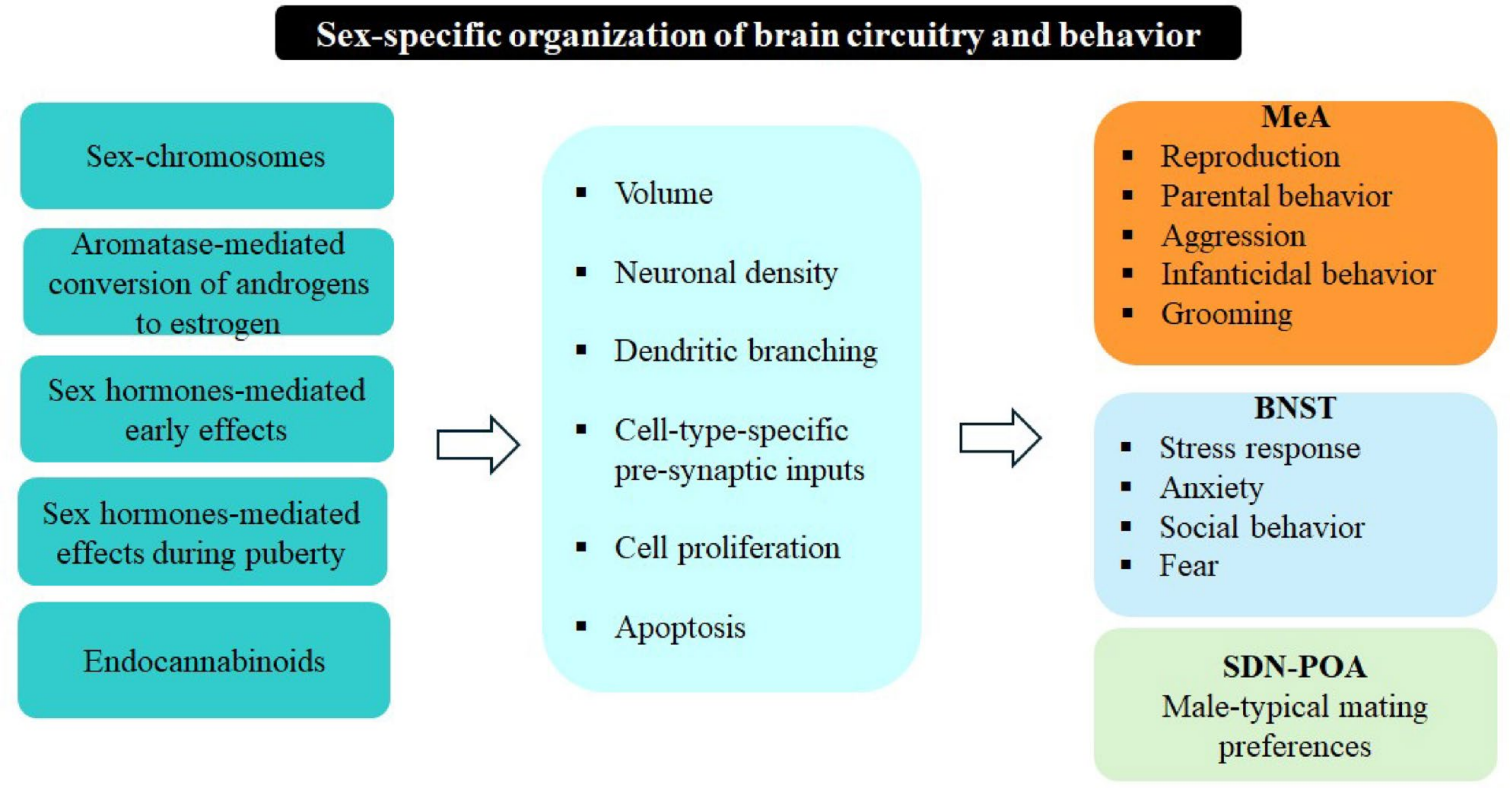
Sex-specific organization of brain areas such as MeA, BNST, and SDN-POA mediated by sex chromosomes, sex hormones, and endocannabinoids results in sex-typical social and emotional behaviors

In addition to the MeA, BNST, and SDN-POA, studies suggest that other brain regions, such as the lateral septum and the anteroventral periventricular nucleus, show sex-specific organization and play robust sexually dimorphic roles [107–109]. Thus, plasticity in several brain areas encompassing changes in volume, neuronal density, dendritic and axonal plasticity, cell proliferation, and apoptosis, primarily driven by the organizational effects of sex chromosomes and sex hormones as well as the activational effects of sex hormones, regulates social and emotional behaviors in females and males (Fig. 4).

## Concluding remarks

Studies show that sex chromosomes and sex hormones play a critical role in shaping sex differences in behavior. Sex differences in the recruitment of neuronal subtypes during behavior might be a key mechanism underlying sex-typical behavior. Sex-specific modulation of pre- and postsynaptic function, local GABAergic inhibition, membrane excitability, and structural plasticity might be key to sex differences in the recruitment of neuronal subtypes during behavior. Similarly, sex differences in the trafficking and expression of neurotransmitter receptors and ion channels might result in sex-typical synaptic and neuronal activation, leading to selective engagement of brain circuits and behavioral differences between females and males. In addition, sex-specific organizational effects on brain circuitry, resulting in sex differences in neuronal density, dendritic branching, and connectivity, could underlie sex-specific behaviors. The activational effects of sex hormones might further enhance sex-typical effects of brain regions that undergo sex-specific organization. Recent progress in understanding sex-typical synaptic, cellular, and circuit mechanisms provides a strong basis for future research to deepen our knowledge of the underlying causes of sexually exclusive and dimorphic behaviors, as well as the magnitude-based differences in shared behaviors. Additionally, future studies exploring sex-specific nervous system mechanisms such as neuroimmune interactions, neuroendocrinology, metabolic factors, and interactions with peripheral physiology, including the gut microbiome and the gut-brain axis, will significantly improve our understanding of sex-typical behaviors. These advances, primarily through animal studies, are expected to facilitate evidence-based approaches to human conditions given the substantial evidence for sex-typical vulnerabilities to nervous system disorders, such as autism spectrum disorders, attention deficit hyperactivity, affective and anxiety disorders, neuroinflammatory disorders, pain, and neurocognitive disorders. Studying sex differences in cellular, synaptic, and circuit mechanisms in animal models of central nervous system disorders is expected to provide critical insights into sex-typical vulnerabilities to these disorders. Understanding how sex differences in cellular, synaptic, and circuitry functions develop with age may be key to learning about sex-specific patterns in neurodegenerative and neurocognitive disorders. Therefore, future multidisciplinary efforts to uncover sex-specific mechanisms in the nervous system will be crucial for understanding sex-dependent behaviors and for informing the development of therapeutic strategies for nervous system disorders.

## Data Availability

No datasets were generated or analysed during the current study.

## Abbreviations

AMPA: Alpha-amino-3-hydroxy-5-methyl-4-isoxazolepropionic acid
AOB: Accessory olfactory bulb
BNST: Bed nucleus of the stria terminalis
CRH: Corticotropin-releasing hormone
GABA: Gamma-aminobutyric acid
LTP: Long-term potentiation
MeA: Medial amygdala
NMDA: N-methyl-D-aspartate
SDN-POA: Sexually dimorphic nucleus of the preoptic area
VGLUT3: Vesicular glutamate transporter 3
VNO: Vomeronasal organ
VTA: Ventral tegmental area
